# Finite element and in vitro study on biomechanical behavior of endodontically treated premolars restored with direct or indirect composite restorations

**DOI:** 10.1038/s41598-022-16480-0

**Published:** 2022-07-25

**Authors:** Tatjana Maravić, Allegra Comba, Claudia Mazzitelli, Luca Bartoletti, Irene Balla, Elisabetta di Pietro, Uroš Josić, Luigi Generali, Darko Vasiljević, Larisa Blažić, Lorenzo Breschi, Annalisa Mazzoni

**Affiliations:** 1 Department of Biomedical and Neuromotor Sciences, DIBINEM, University of Bologna - Alma Mater Studiorum, Via San Vitale 59, Bologna, 40125 Italy; 2 Faculty of Medicine Novi Sad, University of Novi Sad, Hajduk Veljkova 2, Novi Sad, 21000 Serbia; 3 Department of Surgical Sciences, University of Turin, Via Nizza 230, Turin, 10126 Italy; 4 Clinic for Pediatric and Preventive Dentistry, School of Dental Medicine, University of Belgrade, Dr Subotica 15, Belgrade, 11000 Serbia; 5 Unit of Dentistry and Oral-Maxillo-Facial Surgery, Department of Surgery, Medicine, Dentistry and Morphological Sciences, University of Modena and Reggio Emilia, Via del Pozzo 71, Modena, 41125 Italy; 6 Instutute of Physics, University of Belgrade, Pregrevica 118, Belgrade, 11000 Serbia

**Keywords:** Dental diseases, Computational models, Image processing

## Abstract

Objectives of the study were to investigate biomechanical properties of severely compromised premolars restored with composite restorations using finite element analysis (FEA), and in vitro fracture resistance test. A 3-D model of an endodontically treated premolar was created in Solidworks. Different composite restorations were modelled (direct restoration-DR; endo-crown-EC; post, core, and crown-C) with two different supporting tissues: periodontal ligament/alveolar bone (B), and polymethyl methacrylate (PMMA). Models were two-point axially loaded occlusally (850 N). Von Mises stresses and strains were calculated. The same groups were further tested for static fracture resistance in vitro (n = 5, 6.0 mm-diameter ball indenter, vertical load). Fracture resistance data were statistically analyzed (*p* < 0.050). The highest stresses and strains in all FEA models were observed on occlusal and vestibular cervical surfaces, corresponding to fracture propagation demonstrated in vitro. C showed the lowest stress in dentin, while EC showed lower stresses and strains in crown cement. B models demonstrated larger high stress areas in the root than PMMA models. No significant differences in fracture resistance (N) were observed between groups (DR: 747.7 ± 164.0, EC: 867.3 ± 108.1, C: 866.9 ± 126.3; *p* = 0.307). More conservative restorations seem a feasible alternative for endodontically treated premolars to conventional post-core-crown.

## Introduction

Endodontic therapy is usually indicated as a consequence of an extensive carious process or dental trauma, both leading to substantial tooth tissue loss^1,2^. Tissue loss has been appointed the main cause of deterioration of the biomechanical properties of endodontically treated teeth, particularly the loss of proximal ridges and access cavity preparation^2–5^. The definitive restoration of an endodontically treated tooth is of utmost importance for tooth survival and for the restitution of its biomechanical properties^2^. Traditionally, metal-ceramic or all-ceramic full crowns have been used, often with a post as an additional element of retention^6^. Clinically, several studies reported a positive impact of post placement on the survival of endodontically treated premolars^7,8^. This is particularly the case in teeth that present a ferrule effect, but no coronal walls^9^. Hence, to comply with the tendency for procedural simplification and tooth tissue preservation, the application of a post in teeth that exhibit coronal walls should be carefully reconsidered^9^. Nowadays, in the era of minimally invasive dentistry, there has been a shift towards less invasive restorative solutions—Prevention of Extension instead of Extension for Prevention^10^. Resin composites and adhesive systems have improved immensely since their first introduction to the dental field^11,12^ and can provide similar fracture resistance^13–15^ and clinical survival^16,17^ compared to indirect ceramic restorations. The advantage of composite restorations is their repairability and lower cost compared to the ceramic ones.

CAD/CAM indirect restorations are chair-side solutions which reduce laboratory time and costs and are becoming increasingly present in daily clinical practice^18^. Particularly, endocrown, introduced more than two decades ago^19^ has shown promising results in terms of durability and ease of manufacturing and seems to be comparable to full crown restorations in terms of teeth survival rates and fracture resistance, especially in molars^20,21^. However, endocrowns seem to fail more frequently in premolars^22^.

Hence, it is important to further investigate the causes underlying the more unpredictable clinical performance of endocrowns in premolars compared to molars. Due to the complexity and ethical issues of clinical studies, it is advisable to first retrieve in vitro and FEA data on a certain topic. FEA is a method widely used in dentistry that provides a vast array of possibilities to test materials and restorative options, that can be, to a great extent, extrapolated to the clinical setting^23,24^. In order to mimic the biomechanical behavior of dental tissues, the periodontal ligament (PDL) and alveolar bone are simulated in the FEA. A recent review of FEA studies validated using in vitro studies demonstrated that there is a discrepancy between the design of the FEA study and the in vitro validation in terms of supporting tissue modeling^25^. In the FEA, alveolar bone and/or PDL were simulated, while in the in vitro studies teeth were embedded in epoxy resin, composite resin, or silicone^25^. This could have introduced a significant amount of variability to the results of the studies. However, up to the authors’ knowledge, this important matter has not been addressed in the literature.

Therefore, the aim of the present study was to investigate the von Mises stresses and equivalent strains in an endodontically treated upper second premolar with no remaining coronal walls restored using: (a) DR, (b) CAD/CAM EC, or (c) CAD/CAM C by means of FEA, and to validate the 3-D model of an endodontically treated upper second premolar using an in vitro static fracture test. A further aim was to investigate whether supporting tissue modelling (alveolar bone and PDL (B), or PMMA) influences the results of the FEA studies. The null hypotheses were that: (1) the type of restoration does not affect von Mises stress and equivalent strain values in the restorative materials or dental tissues; (2) the type of restoration does not affect von Mises stress and equivalent strain distribution in the restorative materials or dental tissues; (3) supporting tissue modelling does not affect von Mises stress and equivalent strain values in the restorative materials or dental tissues; (4) supporting tissue modelling does not affect von Mises stress and equivalent strain distribution in the restorative materials or dental tissues.

## Results

### FEA

#### Maximum von Mises stress values

The maximum von Mises stresses in the investigated models are presented in Table 1. The maximum stresses in the restorations were found to be similar in all the groups, while in the dental tissues, certain differences were evident among the groups. The stresses in dentin were lower in the indirect restorations compared to the direct restoration (DR > EC > C) regardless of the supporting tissue used. The stresses in the enamel were higher in the EC compared to DR, and slightly higher in all the models with the PMMA support. In the root portion of the C model, higher stresses were found in dentin and the restorative materials in the B supported model, compared to the one embedded in PMMA.

**Table 1:** Maximum Von Mises stresses (MPa).

	Direct restoration		Endocrown		Post, core and full crown	
	Bone	PMMA	Bone	PMMA	Bone	PMMA
Crown restoration	735.3	735.4	736.1	736.0	731.1	731.1
Dentin	125.1	127.0	113.3	117.4	90.3	86.1
Enamel	230.6	236.4	256.0	261.6	–	–
Crown cement	–	–	38.5	38.6	60.2	62.8
Post	–	–	–	–	66.6	50.7
Post cement	–	–	–	–	27.5	18.0
Composite build-up	–	–	–	–	38.3	38.3

#### Von Mises stresses distribution

**Figure 1:**
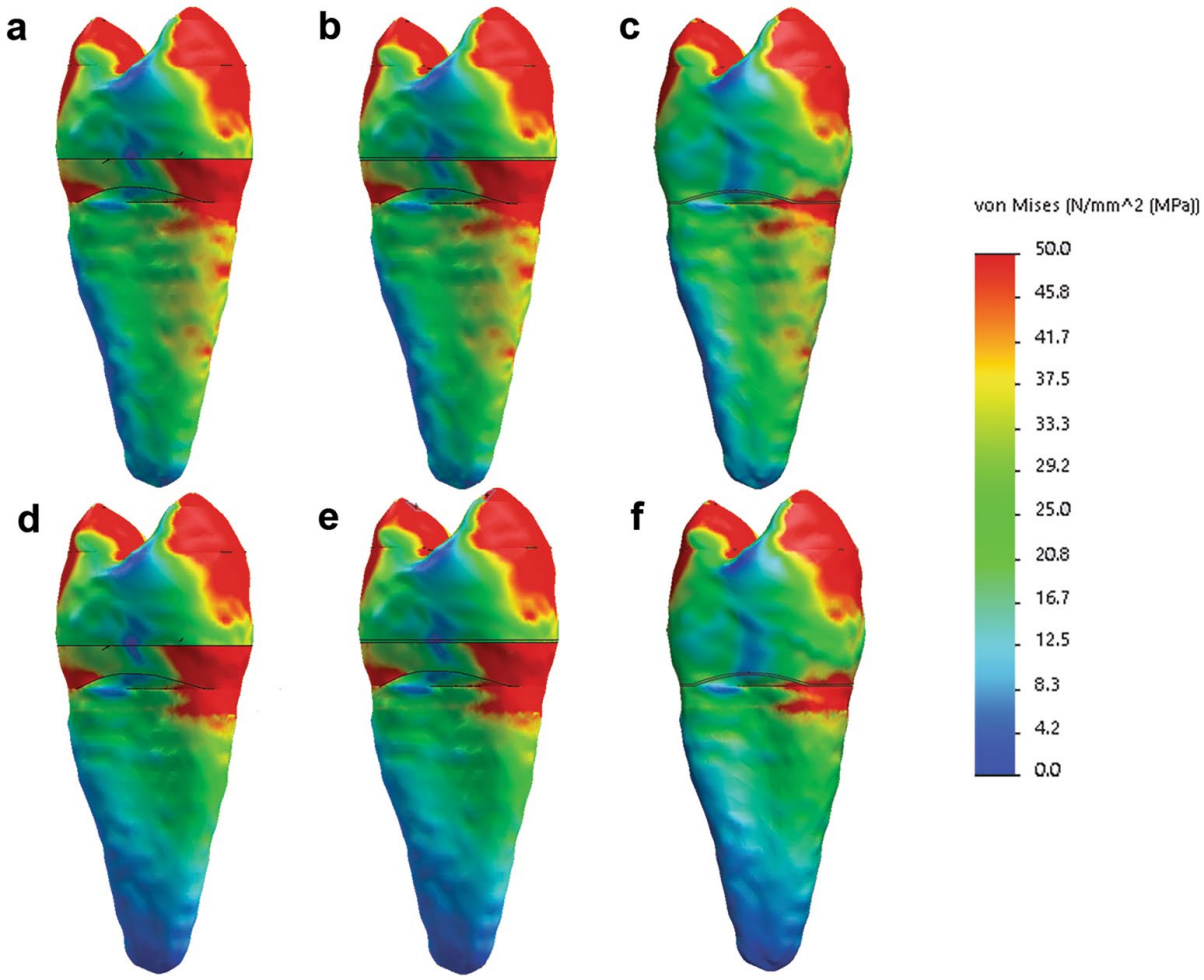
Von Mises stresses distribution: (**a–c**) models restored with DR, EC, and C, respectively, with periodontal ligament and bone as supporting tissue; (**d**–**f**) models restored with DR, EC, and C, respectively, with polymethyl methacrylate as supporting tissue (Solidworks 2014; available at: https://www.solidworks.com/).

**Figure 2:**
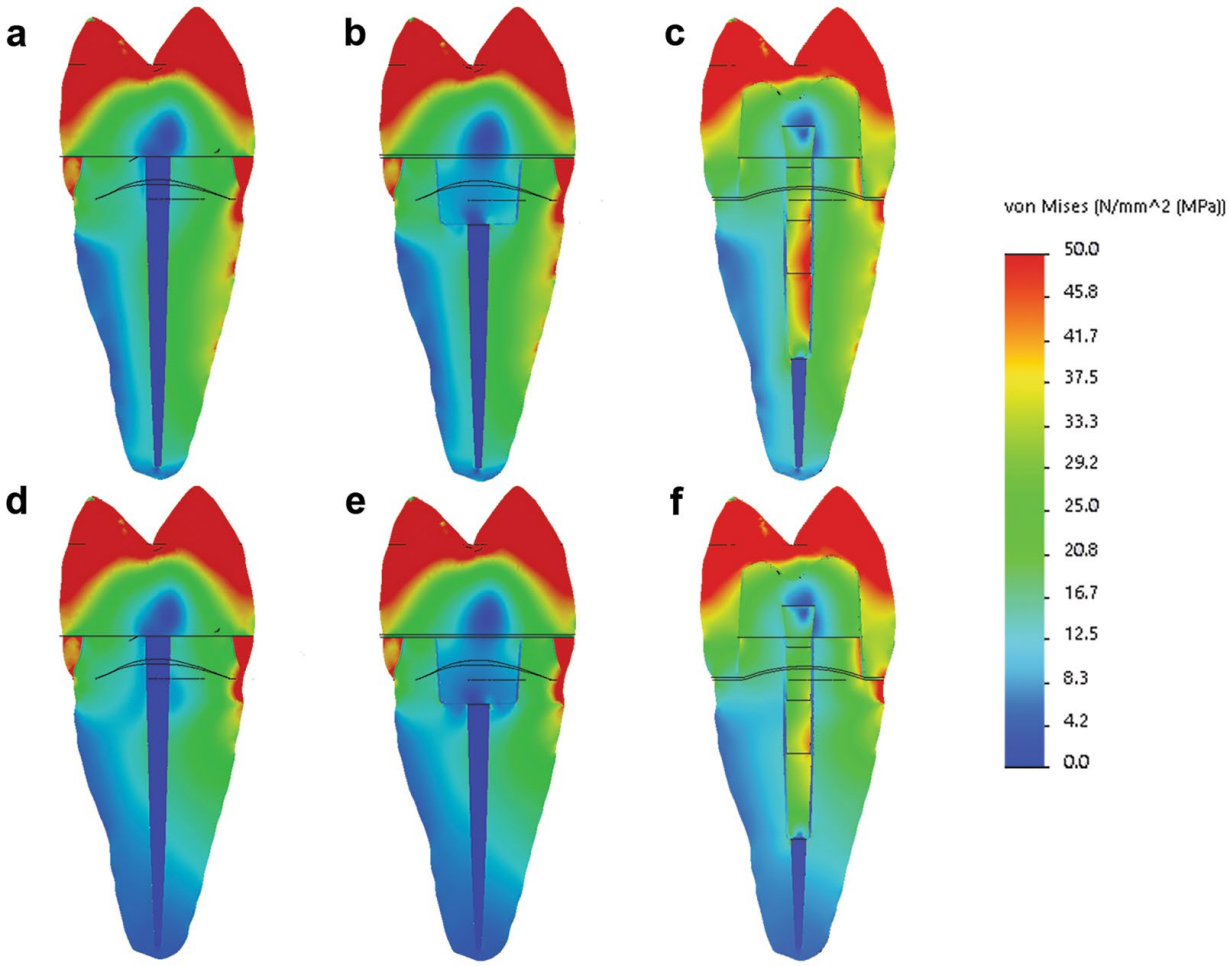
Von Mises stresses distribution - cross-section of the models: (**a**–**c**) models restored with DR, EC, and C, respectively, with periodontal ligament and bone as supporting tissue; (**d**–**f**) models restored with DR, EC, and C, respectively, with polymethyl methacrylate as supporting tissue (Solidworks 2014; available at: https://www.solidworks.com/).

The highest stress areas were distributed similarly in the crowns of all the tested models: on both cusps, on the occlusal central fissure, as well as on the cervical segment of the enamel and dentin, particularly the vestibular portion. In the DR and EC models, the stress distribution was similar, however, there were certain differences between these two restoration types and the C models. Although the occlusal stress distribution in all the models was similar, the crown-restored models showed a slightly smaller area under high stresses in the cervical vestibular segment (Figs. 1, 2).

**Figure 3:**
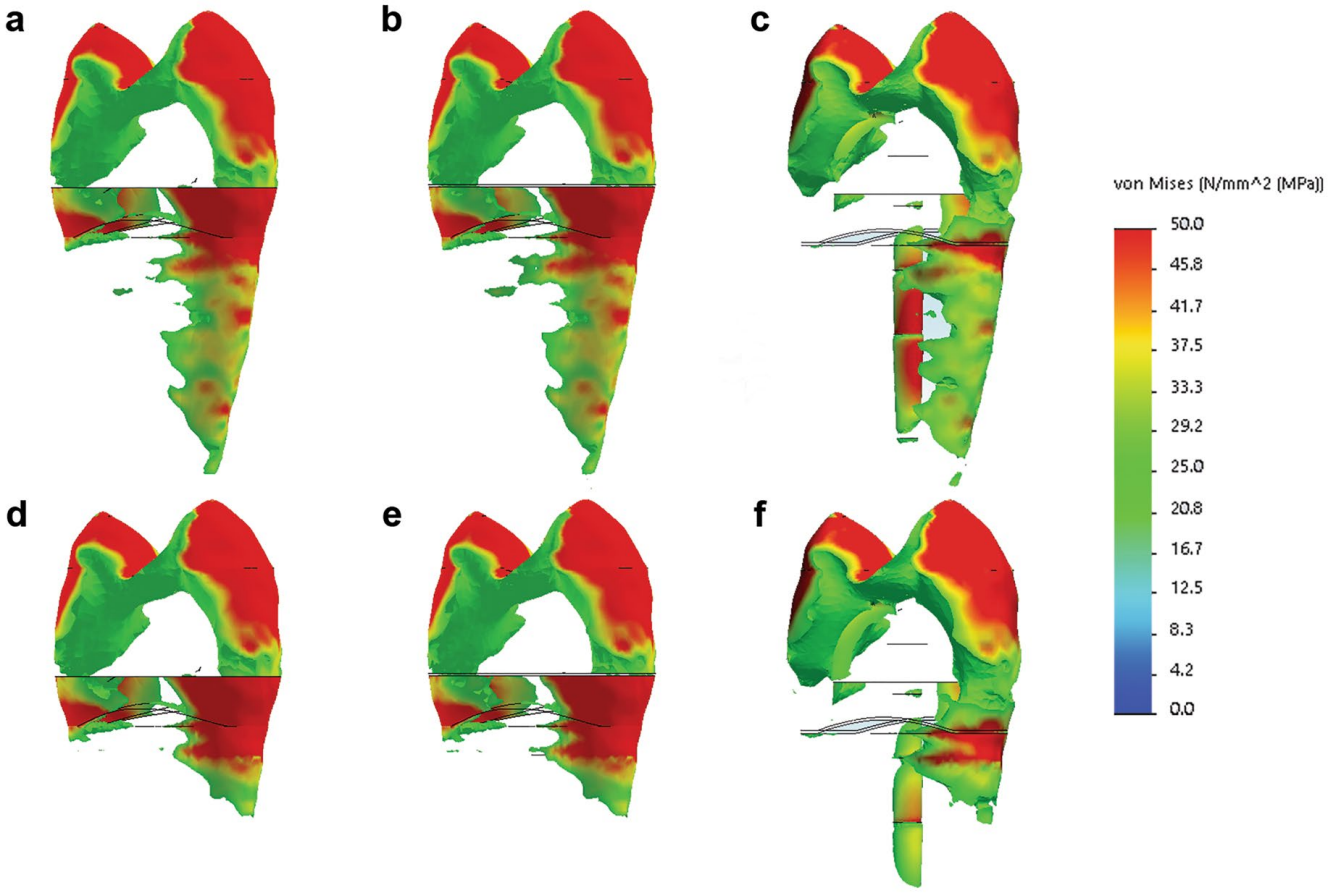
Higher stress areas in the investigated models: (**a**–**c**) models restored with DR, EC, and C, respectively, with periodontal ligament and bone as supporting tissue; (**d**–**f**) models restored with DR, EC, and C, respectively, with polymethyl methacrylate as supporting tissue (Solidworks 2014; available at: https://www.solidworks.com/).

Interestingly, although the maximum stress values were similar, the distribution of stresses was different between the groups having B as supporting tissue, compared to the PMMA. There were larger areas of high stresses in the root portion of all the models supported by B (Fig. 3). The most prominent differences can be noted in the CB model, where the highest stresses in dentin were not distributed at the cervical vestibular portion of the root as in the CPMMA and all the other investigated models, but were located on the bottom of the post cavity preparation.

#### The von Mises equivalent strains

The maximum values of von Mises equivalent strains in each constituent of each model are shown in Table 2. The strains were the highest in the crown restorations of all models, regardless of the supporting tissue simulation option. All the investigated models had similar crown restoration strain values. Similarly to the stresses, the strains in dentin were to the most part comparable in the DR and EC models, while they were lower in the C models. Interestingly, the strains in enamel in the EC groups were 3 times lower compared to the DR groups. EC also showed ~ 35% lower strains in the crown cement compared to C groups. Further, there were differences in the strain values between the CB and CPMMA groups in the root portion of the model, with the CB group demonstrating higher strains.

**Table 2:** Equivalent von Mises strain.

	Direct restoration		Endocrown		Post, core and full crown	
	Bone	PMMA	Bone	PMMA	Bone	PMMA
Crown restoration	0.05883	0.05884	0.05394	0.05394	0.05399	0.05399
Dentin	0.004501	0.004578	0.004452	0.004538	0.004065	0.003327
Enamel	0.004501	0.004578	0.001494	0.001508	–	–
Crown cement	–	–	0.004671	0.004662	0.006974	0.007185
Post	–	–	–	–	0.001179	0.0009019
Post cement	–	–	–	–	0.003413	0.002185
Composite build-up	–	–	–	–	0.004363	0.004364

The maximum strain values were distributed in the same manner as the maximum stresses. Hence, the highest strain in the CB model in dentin was located on the bottom of the post preparation, while in the CPMMA model it was on the cervical vestibular portion of the root, similarly to all the other models.

### Static fracture resistance test: in vitro validation

The results of the one-way ANOVA test presented in Table 3 showed no statistically significant differences in the static fracture test values between the three tested groups (*p* = 0.307).

**Table 3:** Fracture resistances of the groups expressed in Newton and failure modes of samples.

	Mean fracture resistance (± standard deviation, given in N)	Non-restorable fractures (%)	Restorable fractures (%)
DR	747.7 (± 164.0)	60	40
EC	867.3 (± 108.1)	100	0
C	866.9 (± 126.3)	80	20

**Figure 4:**
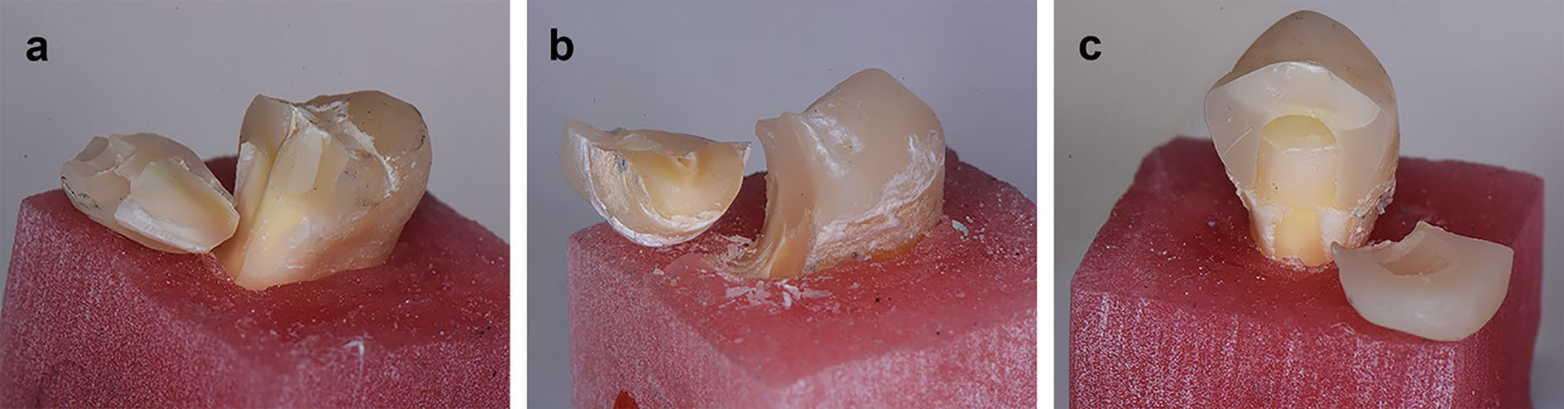
Failure modes of the fractured teeth: (**a**) DR; (**b**) EC; (**c**) C.

The evaluation of the fracture modes was performed by three different evaluators using a stereomicroscope. The agreement between the evaluators was 100%, and the results showed that in all the teeth restored with an EC fractures were unrestorable, followed by 20% and 40% of restorable fractures in the C and the DR groups, respectively (Table 3, Fig. 4).

The failure of the teeth in the in vitro study occurred in the portion of the teeth that corresponded to the high stress areas of the FEA model—started on the occlusal surface on the inner slope of the buccal cusp near the central fissure and propagated towards the vestibular cervical portion of the tooth.

## Discussion

The results of the present FEA study demonstrated differences in the maximum von Mises stress and equivalent strain values in dental tissues and restorations within the investigated groups depending on the restoration type and the supporting tissue modeling. Hence, the first and third null hypotheses were rejected. Further, the type of restoration as well as the modeling of the conditions surrounding the tooth induced differences in the distribution of the maximum stresses in dentin, with possible implications in the clinical setting. Therefore, the second and fourth null hypotheses were also rejected.

Maximum von Mises stresses and their distributions were similar in the coronal restorations of all the FEA models investigated in the present study, while strains were slightly lower in the indirect restorations. This corresponds to the results of the fracture resistance test, since there were no statistically significant differences between the tested groups although the mean fracture resistance was lower in the DR group. These results are in accordance with several FEA and in vitro studies^14,26,27^, while certain authors reported higher fracture resistance and lower stresses in premolars restored with endocrowns compared to the conventional crowns^28,29^. Contrary to this, another study^30^ showed lower survival rate of premolars restored with endocrowns.

While the previously mentioned reports available in the literature^14,26–30^ are contradictory, a more detailed analysis of the interplay between stress and strain values and distributions in the models of the present study could offer interesting inputs. Firstly, crown-restored models showed lower von Mises stresses and equivalent strains in dentin compared to the DR and EC. Although this could lead to the conclusion that the post, core and crown restoration could clinically show better preservation of the dental tissues, note should be taken of the location of the maximum stresses. In the CB group, maximum von Mises stresses in dentin could be noted on the bottom of the post preparation cavity, rather than on the vestibular cervical portion of the tooth as in the other investigated groups. It seems that the post absorbed a certain amount of the stresses, but also transported them to the root portion of the tooth, and could hence potentially act as a wedge leading to catastrophic tooth fracture, as demonstrated in vitro^31^. This is in accordance with the report of a reduced stress concentration on the inner wall of the root in premolars restored with an endocrown compared to a post, core and crown restoration^32^. In general, post, core and crown restorations tend to undergo catastrophic root fractures more often compared to endocrowns^22^. Actually, it was demonstrated clinically that post placement can reduce the failure of post-endodontic restorations only within severely compromised teeth (when no coronal walls are present), and might therefore not be necessary in teeth with some of the tooth tissues preserved^9^. These affirmations are valid for traditional FRC posts, but customized posts have emerged in recent years, and could potentially offer better retention of the coronal restoration, while preserving the anatomy of the root canal and tooth tissue. It was demonstrated in vitro that there could be benefits to the use of auxiliary posts and/or composite resin relined posts in terms of fracture strength and failure pattern, as well as bond strength to root dentin, possibly due to a reduction in the thickness and defects of the cement layer^33–35^. FEA generated stress distribution in incisors showed a more favorable pattern compared to traditional systems^36^, but FEA studies in premolars on this issue are currently lacking.

Further, in comparison to the crown cement in the C models, the cement of the EC models demonstrated around 35% lower von Mises stresses and strains, probably due to the geometrical differences between these two types of crowns. Endocrown is a more massive monolithic restoration, protecting the underlying cement layer from the direct influence of the occlusal loads, as also shown previously^37^. This could indicate that the full crowns may be more prone to debonding compared to endocrowns. Moreover, stresses in the enamel of the DR models were slightly lower than that of the EC models. However, the strains were 3 times lower in the enamel of the EC models. Actually, the crown cement layer in the EC models showed a similar maximum strain value as the enamel in the DR models. Hence, it seems that the cement layer “buffered” the strain of enamel under the endocrown, possibly due to the lower elastic modulus compared to both the crown and the enamel, which could lead to better preservation of the enamel tissue during fatigue loading in teeth restored with endocrowns compared to direct restorations^3^.

The majority of samples in all groups failed in a similar manner in the static fracture test, with the crack initiation at the inner slope of the buccal cusp, near the central occlusal fissure, and the fracture most often reaching the cervical vestibular region of dentin under the cement-enamel junction (CEJ). This failure mode corresponds to previously published studies^38–41^. The distribution of high stresses found in the present FEA study in the models embedded in PMMA (under a load that corresponded to the mean fracture load in the static fracture resistance test), revealed a distribution which is in accordance with the fracture propagation of the in vitro study. The areas under the highest stresses were the loading positions on the inner slopes of the cusps, the central fissure, and the cervical vestibular portion of the tooth tissues. There were larger areas of high stresses in the models restored with the DR and the EC compared to the C restored model. Interestingly, the distribution of high stress areas in dentin changed when the PDL and the supporting bone tissue were modelled. There were larger areas under medium level stresses in the root portion of the teeth, and in the C model, the highest stress area moved from the vestibular cervical portion to the bottom of the post preparation cavity. This could further imply (along with the maximum von Mises stress and strain values) that clinically, the post could cause a wedge and lead to root fracture^22,31^. A recent review on validated FEA studies in dentistry reported that although the teeth in the in vitro validation experiments were mostly embedded in epoxy resin, composite resin, or silicone, in the corresponding FEA study, PDL and/or bone were modelled^25^. Hence, up to the authors’ knowledge, this is the first study to investigate both supporting tissue options, that would correspond to the in vivo, as well as in vitro experimental setting.

Clinically, full crown restorations have similar failure rates in premolars and molars^22,30,42^. With the development of the minimally invasive dentistry concept, the preservation of tooth tissue has become a matter of utmost priority^2,43^. Hence, direct restorations and restorations such as endocrowns, modelled to preserve healthy tooth tissues and yet keep the retentive form, have been developed and promoted in the recent years. However, clinically, endocrowns seem to fail at a higher rate in premolars compared to molars^30^, even though these differences are not always statistically significant^16^. Restoration of endodontically treated premolars is challenging due to their morphology and specific position in the tooth arch. Premolars are exposed to more elevated loads than anterior teeth, both in the axial and shear directions, but have a smaller crown and steeper cusps compared to molars and are therefore more fragile, especially after a large portion of the tissue is lost^28,44,45^. Further, the pulp chamber of premolars, providing retention to the endocrown, is considerably smaller compared to molars.

A recent systematic review on the clinical performance of endocrowns in premolars demonstrated that this type of restoration preforms equally well as full crowns in FEA and in vitro studies, but not in a clinical setting^22^. This could be due to several factors. Firstly, the static fracture that is commonly used in the in vitro studies is unlikely to occur in the patient’s mouth. The failure of restorations during intraoral use is nearly always due to fatigue^46,47^. Accordingly, it was demonstrated that bond strengths of posts to root dentin were significantly influenced by thermal^48^ or thermomechanical aging^49,50^, which was not considered in the present research. Further, as demonstrated in the present study, the distribution of the stresses within the tooth-restoration complex is influenced by the supporting tissue modeling, and in vitro studies cannot fully replicate the intraoral setting, nor can they replicate the actual tooth loading conditions during mastication. The static FEA studies on the other hand, allow for a more accurate replication of the intraoral conditions regarding the supporting tissue and tooth loading conditions. However, apart from not accounting for model fatigue, they also usually omit another clinically important factor—operator sensitivity of the adhesive techniques, since perfect bonding is assumed, the Holy Grail not easy to achieve clinically^51,52^. Bonding to root dentin is even less predictable compared to the coronal dentin^53^. It was shown that the currently available luting agents cannot hermetically seal the endodontic cavity^54^. Nevertheless, by simulating perfect bonding, FEA allows researchers to focus on a specific factor that needs to be investigated without interference of other factors. Conversely, the results of in vitro studies can be affected by the experience of the operator and the natural variability of dental tissues, while clinical studies are prone to selection, performance, detection, attrition and reporting bias.

In the present study, we opted to evaluate the Von Mises criterion, a scalar stress measure combining three principal stress values, identifying the areas of the model that are under highest stress and are consequently more prone to failure. This criterion enabled us to evaluate whether the critical areas identified in FEA matched the localization of fracture initiation and propagation of the in vitro specimens, as well as to compare our results with other published studies that used the same criterion^37,39^. Indeed, the calculation of principal stresses could provide a more detailed analysis of the tensile, compressive and shear stress components, and this could be considered a limitation of our study. Further, the sample size of the in vitro section of the present study (n = 5) could be considered low for mechanical testing. However, we performed a sample size calculation and used the recommended sample size. The in vitro part of the study was not performed as a standalone test, but as a complementary experiment to validate whether the propagation of fracture coincides with the stress and strain values and distribution that was demonstrated in the FEA, the main focus of the present study. Moreover, numerous published FEA studies used as much as 4 or 5 teeth for in vitro validation^55–58^.

We can conclude that, in terms of response to static axial loads, direct composite restoration, composite endocrown, as well as post, core and crown seem to be adequate for the restoration of endodontically treated premolars with a severe tissue loss. When a 2 mm ferrule is present, endocrowns demonstrated certain advantages in terms of stresses and/or strains values and distribution in tooth tissues and in the cement layer compared to the other restoration types. Furthermore, differences in the simulation of the supporting tissues can influence the results of FEA studies and should be taken into consideration, particularly when employing a FEA and in vitro experiment in the same study.

## Methods

### FEA

**Figure 5:**
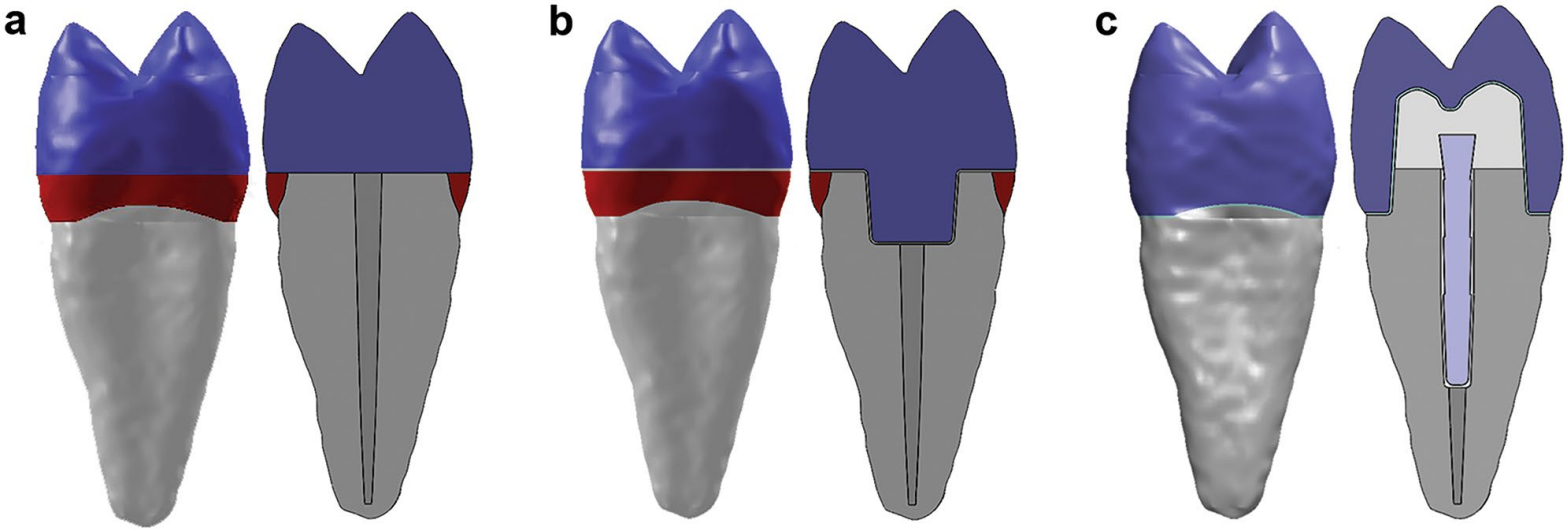
Different restorative options modelled for the FEA, whole tooth, and a cross-section, respectively: (**a**) DR; (**b**) EC; (**c**) C (Solidworks 2014; available at: https://www.solidworks.com/).

The 3-D tooth model used in the present study was created based on a computed tomography scan (Sensation 64 Cardiac CT scanner; Siemens, Munich, Germany) of a sound upper second premolar, extracted for orthodontic reasons following the informed consent of the patient. The research protocol was approved by the Ethical Committee of the University of Bologna (Italy; protocol N°: 71/2019/OSS/AUSLBO). Research was performed in accordance with the Declaration of Helsinki. The detailed model generation procedure was described elsewhere^3,4^. In brief, there were 42 sections in the z-axis that represented tooth tissues and were transferred to a segmentation software (Amira; Thermo Fisher Scientific, Waltham, MA, USA) in the DICOM format. The segmentation was performed based on the differences in the signal density of different tooth tissues. The contours of the tooth tissues were refined and imported to the modelling software (Solidworks 2014; Dassault Systèmes SolidWorks, Waltham, MA, USA), where the solid bodies of enamel, dentin and pulp were created. After the creation of the basic tooth model, a transversal tooth section 2 mm above the CEJ was simulated (ferrule effect), followed by endodontic treatment with rotary instruments (size 25 and taper of 0.6 of the endodontic instrument was simulated) and gutta-percha filling. Further, three different restorative options were created (Fig. 5): Group 1 (DR, control): Direct composite restoration without a retentive cavity; Group 2 (EC): CAD/CAM composite endocrown with a 3 mm-deep intracanal portion and a 100 µm-thick layer of resin cement between the crown and the tooth tissues; Group 3 (C): A glass fiber composite post inserted 8 mm into the tooth canal, composite core, and CAD/CAM composite crown.

Both around the post and underneath the crown, a 100 µm-thick layer of resin cement was modeled. For each of the groups, 2 mm below the level of the CEJ, two different supporting tissue options were modelled: (a) PDL, 0.2 mm thickness and alveolar bone (B); (b) PMMA. Hence, the final analysis included 6 different models: DRB, DRPMMA, ECB, ECPMMA, CB and CPMMA.

Materials were considered to be linear, elastic and isotropic, and material properties were assigned to all the tooth tissues and materials (Table 4).

**Table 4:** Material properties.

Material	Young’s modulus (MPa)	Poisson’s ratio	References
Enamel	84,100	0.20	^59^
Dentin	18,600	0.31	^59^
Periodontal ligament	70	0.45	^59^
Gutta-percha	100	0.49	^60^
Alveolar bone	15,000	0.30	^59^
ParaCore (Coltène/Whaledent, Altstätten, Switzerland)	7500	0.33	*
Composite resin blocks CAD/CAM Brilliant Crios (Coltène/Whaledent)	10,300	0.30	*
DuoCem (Coltène/Whaledent)	6500	0.33	*
ParaPost Taper Lux (Coltène/Whaledent)	45,000	0.30	*
Synergy D6 (Coltène/Whaledent)	9700	0.30	*
Polymethyl methacrylate	2770	0.35	†

*Acquired from the manufacturer.

^†^Acquired from the Solidworks materials library.

In the present study, an axial load of 850 N was applied to 2 points, on the inner slopes of both cusps, to simulate the loading performed in the in vitro fracture resistance testing. The model was fixed in all directions on the outer surface of the bone/bone-simulating PMMA. Perfect bonding between the parts of the models was assumed. Curvature based high-quality meshing was performed and 136,513–229,599 nodes and 87,467–146,189 elements were obtained. Parabolic tetrahedral solid elements were used for meshing, as they enable higher quality meshing for irregular-shaped objects, such as biological tissues. The maximum element size was 2.30906 mm, minimum element size was 0.230906 mm. There were 0.0606–0.268% of the elements with the aspect ratio greater than 10, with 95–96.5% of the elements having a ratio lower than 3. Consecutively, numerical analysis was performed in the “Simulation” add-in of Solidworks. Von Mises stresses and equivalent strains were calculated and recorded.

### Static fracture resistance test: in vitro validation

The dental materials used in the present study were donated by Coltène/Whaledent (Altstätten, Switzerland), unless stated differently. All the materials have been used by two experienced clinicians (T.M., A.C.), strictly following the manufacturer’s instructions.

Fifteen extracted single-rooted premolars (sample size calculated using G*Power 3.1.9.7 for Windows: effect size f = 1.9191754, α error probability = 0.050, power (1-β error probability) = 0.800) were selected and treated endodontically with rotary instruments up to the file size 25 (Mtwo; Sweden & Martina, Due Carrare, Italy) and obturated with gutta-percha (Mtwo gutta; Sweden & Martina, and Gutta percha bar; Meta Biomed, Mülheim an der Ruhr, Germany). Teeth were further prepared in a standardized way, removing the crown leaving 2 mm of sound dentinal tissue in the cervical area above the CEJ. Premolars were randomly divided into three groups (n = 5) according to the protocol employed for the crown restoration, following the FEA groups:

DR: (control)—Selective enamel etching was performed for 30 s with a 37% phosphoric acid (Etching), followed by adhesive resin (One Coat 7 Universal) application and light-curing for 20 s with a LED curing light (Valo; Ultradent, St Louis, MO, USA). The same curing unit was used in all restorative procedures for all the specimens. Further, a direct resin composite restoration (Synergy D6) was stratified in 2 mm-thick layers and each layer was polymerized for 40 s. The occlusal anatomy was created using a transparent silicone mold (Elite Glass; Zhermack, Badia Polesine, Italy) that was made prior to the treatment of the tooth. The composite was polymerized through the silicone mold for 40 s from each side, then the mold was removed, and the curing procedure was repeated.

EC: Three mm of the gutta-percha endodontic filling was removed from the cervical portion of the tooth to prepare the canal space for the retention of a CAD/CAM composite endo-crown (Block Brilliant Crios; crown wall thickness < 5 mm) fabricated using a milling system (CEREC; Dentsply-Sirona, Charlotte, NC, USA). The luting surface of the crown was sandblasted for 20 s at 1,5 bar pressure with 50 μm particles of sodium bicarbonate (Rondoflex; KaVo Dental, Biberach an der Riss, Germany) washed with water for 20 s, dried with an air flow for 2 s and immerged in an ultrasound bath (Transsonic T460/H; Elma Schmidbaue, Singen, Germany) for 5 min in a 50% ethanol solution (Ethanol; Carlo Erba Reagents, Cornaredo, Italy). Further, an adhesive resin (One Coat 7 Universal) was placed on the composite crown and on the tooth structure. The adhesive was light cured for 20 s only on the tooth surface, and the crown was cemented using a dual cure resin cement (DuoCem). Final light curing step was performed through the crown from each surface of the restoration.

C: The remaining coronal portion of the tooth was prepared for a composite crown (cervical margin of 1 mm, occlusal thickness 1.5–2 mm, 1–1.5 mm axial wall thickness). Teeth were restored with a glass fiber-based composite post (ParaPost Taper Lux), cemented with a dual cured resin-based luting material (DuoCem) 8 mm into the depth of the canal, and polymerized for 30 s using a LED curing unit. Further, a self-etch adhesive system was applied to the tooth structure (Parabond with Non-Rinse Conditioner), followed by a composite build-up (ParaCore Dentin) polymerized for 60 s using a LED curing light. The tooth preparation was finished, and a CAD/CAM composite crown (Block Brilliant Crios) was milled, sandblasted, luted with a universal adhesive (One Coat 7 Universal) and a dual cure resin-based luting cement (DuoCem), and polymerized for 20 s on each side, the same as in the EC group.

Further, lateral static fracture resistance test was performed after 30-days storage in artificial saliva (KCl 0.9639 g/L, KSCN 0.1892 g/L, Na_2_SO_4_·10H_2_O 0.763 g/L, NH_4_Cl 0.178 g/L, CaCl_2_·2H_2_O 0.2278 g/L, NaHCO_3_ 0.6308 g/L, ZnCl_2_ 2.726 mg/L, HEPES 1.186 g/L, pH 7.4) at 37 °C. The roots of the teeth (2 mm under the CEJ) were embedded in methacrylate resin (Impression Tray Resin LC; Henry Schein, Services, Langen, Germany) and the teeth were mounted in a universal test machine (Instron 10-S; Instron, Norwood, MA, USA) at a 45° inclination to the long axis of the tooth. A metal rod with a spherical tip of 6.0 mm diameter was used to apply a vertical static load at a crosshead speed of 0.5 mm/min until the fracture of the specimens occurred.

Further, fractographical analysis of the failure areas was performed under a stereomicroscope (Stemi 2000-C; Carl Zeiss Jena, Germany) at a 30× magnification. The fractures that involve the CEJ or the tooth structure bellow the CEJ were considered unrestorable, while the fractures above the CEJ were considered restorable.

Since the normality (Shapiro–Wilk test), and homoscedasticity assumptions (modified Levene test) were not violated, the data were statistically analyzed using a one-way ANOVA and post-hoc Bonferroni tests with the significance level set at α = 0.050 (Stata; StataCorp, College Station, TX, USA).

## Data Availability

The datasets used and/or analyzed during the current study are available from the corresponding author on reasonable request.
